# Effects of high-intensity interval training on fatigue and quality of life in testicular cancer survivors

**DOI:** 10.1038/s41416-018-0044-7

**Published:** 2018-05-08

**Authors:** Scott C Adams, Darren S DeLorey, Margie H Davenport, Adrian S Fairey, Scott North, Kerry S Courneya

**Affiliations:** 1 Faculty of Kinesiology, Sport, and Recreation, University of Alberta, Edmonton, AB T6G 2H9 Canada; 2 Department of Surgery, University of Alberta, Edmonton, AB T6G 2B7 Canada; 3 Alberta Urology Institute Research Centre, Edmonton, AB T6G 1Z1 Canada; 4 Department of Oncology, University of Alberta, Edmonton, AB T6G 2R7 Canada; 5 Cross Cancer Institute, Edmonton, AB T6G 1Z2 Canada; 6 Present Address: Department of Medicine, Memorial Sloan Kettering Cancer Center, New York, NY 10017 USA

**Keywords:** Quality of life, Testicular cancer

## Abstract

**Background:**

Testicular cancer survivors (TCS) are at increased risk of cancer-related fatigue (CRF), psychosocial impairment, and poor mental health-related quality of life (HRQoL). Here, we examine the effects of high-intensity interval training (HIIT) on patient-reported outcomes (PROs) in TCS. Secondarily, we explore cardiorespiratory fitness as a mediator of intervention effects and select baseline characteristics as moderators of intervention effects.

**Methods:**

TCS (*n* = 63) were randomly assigned to 12 weeks of supervised HIIT or usual care (UC). PROs included CRF, depression, anxiety, stress, self-esteem, sleep quality, and HRQoL assessed at baseline, post-intervention, and 3-month follow-up.

**Results:**

TCS (median 7 years postdiagnosis) completed 99% of training sessions and achieved 98% of target training intensity. ANCOVA revealed that, compared to UC, HIIT significantly improved post-intervention CRF (*p *= 0.003), self-esteem (*p *= 0.029), and multiple HRQoL domains (ps ≤ 0.05). Effects on CRF (*p *= 0.031) and vitality (*p *= 0.015) persisted at 3-month follow-up. Cardiorespiratory fitness changes mediated CRF and HRQoL improvements. CRF effects were larger for TCS with an inactive lifestyle, lower fitness, higher testosterone, and clinical fatigue at baseline.

**Conclusions:**

HIIT significantly improves CRF and HRQoL in TCS. Mediation by cardiorespiratory fitness and moderation by clinical characteristics suggests opportunities for targeted exercise interventions to optimise PROs in TCS.

## Introduction

In North America, testicular cancer (TC) is the most commonly diagnosed malignancy in men 15 to 35 years^1^. Conventional therapies used to treat TC (i.e., orchidectomy, radiotherapy, and chemotherapy) are highly effective at curing germ-cell tumours^2^ even with advanced disease^3^. Unfortunately, TC and its treatments are associated with adverse health effects including second cancers^4^, cardiovascular disease^5^, peripheral neuropathy^4^, hypogonadism^4^, cancer-related fatigue (CRF)^4,6^, anxiety^7^, poor mental health-related quality of life (HRQoL)^8^, and possibly depression^8^.

Among patient-reported outcomes (PROs), CRF may be especially burdensome for TCS. The National Comprehensive Cancer Network defines CRF as 'a persistent subjective sense of tiredness related to cancer or cancer treatment that interferes with usual functioning'^9^. Fatigue is more prevalent in TCS than in the general population (17–30% vs. 10–12%) and increases from 12–19 years posttreatment, independent of treatment exposure^6,10^. CRF is among the most frequent and distressing symptoms in TCS^11^, and is associated with low testosterone^6^, poor HRQoL^10^, anxiety^6^, depression^6^, and cognitive impairments^4^.

Aerobic exercise training is a promising intervention for improving CRF^12^, depression^13^, and HRQoL^14^ in several cancer survivor groups; however, few studies have focused on TCS. Cross-sectional evidence in TCS suggests that physical activity is associated with lower levels of CRF^6^, depression^15^, and adverse health outcomes^16^. To date, however, no randomised controlled trials have examined whether aerobic exercise training improves PROs that are important to TCS.

The High-Intensity Interval Training in Testicular cancer Survivors (HIITTS) trial was developed to assess the effects of 12 weeks of high-intensity aerobic interval training (HIIT) on cardiovascular disease risk in TCS. In the primary paper, we reported that HIIT significantly improved cardiorespiratory fitness (VO_2peak_), vascular structure and function, parasympathetic nervous system function, and blood-based biomarkers of cardiovascular disease^17^. Here, we report the effects of HIIT on PROs at post-intervention and 3-month follow-up. Based on previous research in other cancer populations^12–14^, we hypothesised that HIIT would improve CRF, psychosocial functioning, and HRQoL compared to usual care (UC). It was unclear if these improvements would be maintained at 3-month follow-up. Moreover, we explored whether VO_2peak_ mediated improvements in PROs. Based on previous research in breast cancer^18,19^ and lymphoma patients^20^, we hypothesised that VO_2peak_ would significantly mediate improvements in the physical and functional components of HRQoL, but not the psychosocial components. Finally, we explored selected baseline characteristics as potential moderators of the effects of HIIT on CRF.

## Patients and Methods

### Settings and participants

HIITTS trial methods have been reported elsewhere^17^. Briefly, participants were recruited through the Alberta Cancer Registry and the surveillance clinic at the Cross Cancer Institute in Edmonton, AB. Men between the ages of 18 and 80 with a confirmed history of stage I-IV TC and who were post-surgery/treatment were eligible. Exclusion criteria: the inability to complete the first two stages of the aerobic exercise test, the presence of any uncontrolled cardiovascular condition, the presence of any psychiatric condition, or the performance of regular vigorous intensity aerobic exercise. The Health Research Ethics Board of Alberta–Cancer Committee (Trial ID# 14-0183) and the University of Alberta approved this trial (Clinical Trial Registration #NCT02459132).

### Design and procedures

The HIITTS trial was a prospective, two-armed, phase II, randomised controlled trial. Eligible participants signed informed consent and completed a baseline aerobic exercise test, resting vascular and nervous system tests, and a self-report questionnaire.

### Randomisation and blinding

Participants were stratified by age (<50 vs. ≥50 years) and treatment exposure (surgery-only vs. any adjuvant therapy) and randomised to HIIT or UC in a 1 to 1 ratio using a variable 4–6 permuted block design. A research assistant, not otherwise involved in the study, generated the allocation sequence via computer-generated random numbers list. It was not possible to blind the participants or interventionists to the group allocation. Outcome assessors were not blinded for the maximal fitness test. Staff blinded to group allocation performed data entry and analysis.

### Exercise training and UC conditions

HIIT participants were asked to attend three supervised exercise sessions per week, consisting of uphill treadmill walking or running, and to maintain all other exercise they were performing at baseline^21^. Each supervised HIIT session was 35 min in length, starting with a 5-minute warm-up [performed within 5% of ventilatory threshold (calculated from the maximal exercise test)^22^], transitioning into the work period, and ending with a 5-minute cool-down. The work period consisted of four, 4-minute, high-intensity intervals. The intensity gradually increased from 75% to 95% of VO_2peak_ over the intervention period. Each 4-minute high-intensity interval was separated by a 3-minute active recovery interval (performed 5% to 10% below ventilatory threshold). Heart rate monitors and logs were used to track exercise adherence, and programs were augmented to maintain target heart rates. Exercise adherence was supported by providing flexible 7-day/week access to the training facility, free parking, and one-on-one exercise session supervision. UC participants were asked to maintain their baseline exercise levels. Each UC participant was informed that, following the 12-week intervention period and 3-month follow-up assessment, they would be offered a free 6-week supervised HIIT program.

### Assessment of participant characteristics

Participant demographic, medical, behavioral, psychosocial, and HRQoL variables were assessed via self-report. Self-directed exercise was assessed at baseline, post-intervention, and at 3-month follow-up using the Godin Leisure Time Exercise Questionnaire^23^. Exercise minutes were calculated as moderate-intensity minutes plus two times vigorous intensity minutes^24^.

### Assessment of patient-reported outcomes

PROs were assessed at baseline (within 48 hours prior to randomisation), immediately post-intervention (HIIT: within 4 days of intervention cessation; UC: after 12 weeks), and at 3-month follow-up. Post-intervention was the primary time point of interest. CRF was assessed using the Functional Assessment of Cancer Therapy Fatigue scale (FACT-F) which is a 13-item inventory assessing 1-week CRF severity on a 0–4 scale with higher scores reflecting lower CRF^25^. Depression was evaluated using the Center for Epidemiologic Studies Depression Scale 10-item inventory which assesses 1-week depressive symptom frequency on a 0–3 scale with higher scores reflecting higher depressive symptom frequency^26^. Anxiety was evaluated using the Spielberger State Anxiety Scale 10-item inventory, which assesses 1-week anxiety symptom severity on a 1–4 scale with higher scores reflecting increased anxiety symptom severity^27^. Stress was evaluated using the Perceived Stress Scale 14-item inventory which assesses 1-month stress symptom frequency on a 0–4 scale, wherein higher scores reflect greater stress symptom frequency^28^. Self-esteem was assessed using the Rosenberg Self-Esteem Scale 10-item inventory using a 1–4 scale, wherein higher scores reflect greater self-esteem^29^. Sleep quality was assessed using the Pittsburgh Sleep Quality Index which assesses 1-month subjective sleep quality with lower scores reflecting better sleep quality^30^.

HRQoL was assessed using the well-validated SF-36^®^^31^. The SF-36 is a 36-item scale assessing eight health domains including physical functioning, role-physical, bodily pain, general health, vitality, social functioning, role-emotional, and mental health. The scores for each subscale were then transformed into norm-based scores wherein higher scores reflect better functioning. The mental component score (MCS) and physical component score (PCS) were calculated by adding the prespecified weighted contributions of each of the eight subscale scores.

### Exploratory analyses

Exploratory mediation analyses were conducted using the product of coefficients method^32^, wherein a series of linear regressions were used to test for possible mediation. We examined the potential mediating role of VO_2peak_ for any PRO that was statistically or borderline significant (*p* < 0.10) at post-intervention and 3-month follow-up. Based on our mediation model (Figure S1), this approach required (1) calculating the total effect of group allocation on the PRO (*path c*), (2) calculating the effect of group allocation on VO_2peak_ (*path a*), (3) calculating the association between VO_2peak_ and the PRO (*path b*), and (4) calculating the direct effect of group allocation on the PRO (*path c’*) while controlling for the indirect effect (product of coefficients *a* × *b*). The statistical test of mediation is the examination of the bias corrected 95% CIs for the mediation effect (*a* × *b*) using a bootstrapping method involving 5000 bootstrap resamples^33,34^.

Our exploratory moderation analyses were conducted using linear regression analysis with simple slopes analyses and interaction tests^33^. Wherever possible, potential moderators were stratified according to clinical cut-points or exposures, as follows: baseline age [younger: <50 years of age, older: ≥50 years of age]; baseline VO_2peak_ [low: <35^th^ percentile, average: ≥35^th^ percentile and ≤65^th^ percentile, and high: >65^th^ percentile of age-/sex-specific values]; baseline aerobic exercise training (AET) [no exercise: not meeting ACSM aerobic exercise guidelines, exercise: meeting ACSM aerobic exercise guidelines]; treatment exposure [no chemotherapy: did not receive chemotherapy, chemotherapy: received chemotherapy]; and baseline fatigue [fatigued: FACT-F score < 42 (clinically fatigued), not fatigued: FACT-F score ≥ 42]. Baseline testosterone was stratified via median split [Low: <14.5 nmol/L, High: ≥14.5 nmol/L]. Given the exploratory nature of these analyses, variables were considered potential moderators if their interaction term *p* values were ≤0.10.

### Assessment of cardiorespiratory fitness

As reported previously^17^, cardiorespiratory fitness (relative VO_2peak_) was assessed at baseline and post-intervention via a treadmill-based maximal cardiorespiratory exercise test (Woodway—4Front, Waukesha, WI) with a constant belt speed (i.e., individualised to each participant) and an increasing incline (i.e., 2% every 2 min until exhaustion). Oxygen consumption (ParvoMedics—TrueOne 2400, Murray, UT) and heart rate (12-lead ECG; Nasiff—CardioCard, Central Square, NY) were measured continuously throughout the test. We defined VO_2peak_ as the highest 15-second average oxygen-uptake value recorded during the test. Ventilatory threshold, determined using the V-slope method^22^, was used to prescribe exercise intensities.

### Statistical analyses and sample size calculation

The primary objective of the trial was to determine the effects of HIIT on VO_2peak_ compared to UC^17^. Based on ANCOVAs adjusting for baseline values and relevant covariates^35^, 66 participants provided 80% power to detect a difference of 3.5 ml O_2_/kg/min, with a two-tailed alpha = 0.05. This level of power translates into a standardised effect size *d* of ~0.60 which applies to the PROs examined in this paper. We report unadjusted baseline, post-intervention, and 3-month follow-up data and adjusted post-intervention and 3-month follow-up data, adjusted between-group mean difference, 95% confidence interval (CI) and *p*-value for all hypothesised comparisons. Data were analysed using an intention-to-treat approach for all participants with post-intervention or 3-month follow-up data (SPSS version 24). Our exploratory mediation and moderation analyses were conducted using the PROCESS plug-in for SPSS^33^.

## Results

### Participant flow

**Fig. 1:**
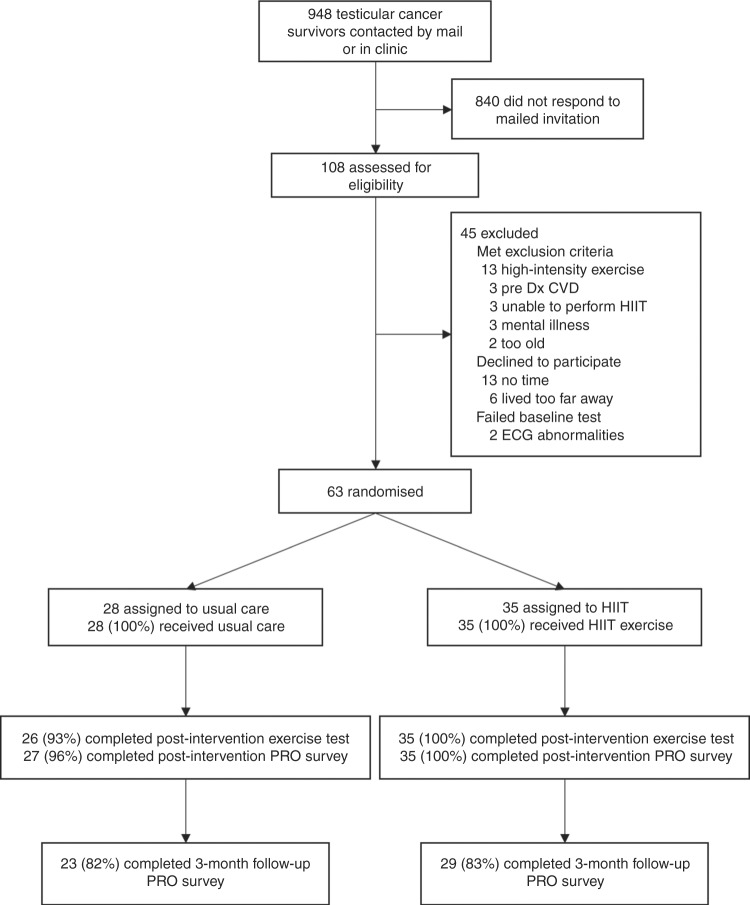
Participant flow through the HIITTS trial. Dx diagnosis, CVD cardiovascular disease, HIIT high-intensity aerobic interval training, ECG electrocardiogram, PRO patient-reported outcome

Participant flow through the study has been reported elsewhere^17^. Briefly, recruitment took place from June 2015 to March 2016 (Fig. 1). Of the 948 potentially eligible participants who were initially contacted, 108 (11%) were screened for eligibility and 63 (7%) were randomised. We obtained post-intervention PRO data on 62 of 63 (98%) participants and 3-month follow-up data on 52 of 63 (83%) participants. No exercise-related serious adverse events were reported or observed.

### Baseline characteristics, participant adherence, and fitness changes

Participant baseline demographics, medical, and behavioral profiles were reported previously^17^ and are summarised in Table 1. Briefly, the participants were on average 43.7 years of age, 92.1% had a single orchidectomy, 36.5% received chemotherapy, and they self-reported an average of 105 exercise minutes/week^23^. The groups were balanced on baseline descriptive variables. Exercise attendance was 99% and participants achieved 98% and 103% of their target heart rates during the work and recovery phases, respectively. During the intervention period, self-directed exercise remained low (123 min/week) and was not significantly different between groups (*p* = 0.37). HIIT improved VO_2peak_ by 3.7 ml O_2_/kg/min (*p* < 0.001) compared to UC^17^. At 3-month follow-up, self-reported exercise increased significantly from baseline (average 263 min; *p* < 0.001), but was not significantly different between groups (*p* = 0.23).

**Table 1:** Baseline demographic, medical, and behavioral profile of HIITTS trial participants, overall and by group assignment

	Overall (*n* = 63)		Usual Care (*n* = 28)		HIIT (*n* = 35)	
	No. of patients	%	No. of patients	%	No. of patients	%
Demographic Profile						
Age, years						
Mean (SD)	43.7 (10.8)		43.3 (9.9)		44.0 (11.6)	
Range	21–61		22–61		21–60	
Caucasian	57	90.5	27	96.4	30	85.7
Completed university	42	66.7	19	67.9	23	65.7
Married	43	68.3	18	64.3	25	71.4
Medical Profile						
Time since diagnosis, years						
Mean (SD)	8.0 (5.5)		7.5 (5.5)		8.5 (5.5)	
Range	1–20		1–20		1–20	
Treatment years						
≤2000	8	12.7	4	14.3	4	11.4
2001–2005	16	25.4	6	21.4	10	28.6
2006–2010	17	27.0	8	28.6	9	25.7
≥2011	22	34.9	10	35.7	12	34.3
Disease stage at diagnosis						
Localised	41	65.1	20	71.4	22	62.9
Advanced	22	34.9	8	28.6	13	37.1
Surgical protocol						
Single orchidectomy	58	92.1	27	96.4	31	88.6
Received radiotherapy	11	17.5	5	17.9	6	17.1
Abdominal radiotherapy exposure	10	15.9	4	14.3	6	17.1
Received chemotherapy	23	36.5	8	28.6	15	42.9
2 cycles of Cisplatin-based therapy	1	1.6	1	3.6	0	0.0
3 cycles of Cisplatin-based therapy	16	25.4	7	25.0	9	25.7
4 cycles of Cisplatin-based therapy	5	7.9	1	3.6	4	11.4
6 cycles of Cisplatin-based therapy	1	1.6	0	0.0	1	2.9
Physical/Behavioral Profile						
VO_2peak_ (ml O_2_/kg/min)						
Mean (SD)	37.0 (6.2)		37.0 (7.2)		37.1 (5.7)	
Low (<35^th^ percentile)	35	55.6	17	60.7	18	51.4
Average (≥35^th^ & ≤65^th^ percentile)	19	30.2	5	17.9	14	40.0
High (>65^th^ percentile)	9	14.3	6	21.4	3	8.6
Testosterone (nmol/L)						
Mean (SD)	14.9 (5.5)		14.6 (4.8)		15.1 (6.1)	
Low (<14.5 nmol/L)	31	49.2	12	42.9	19	54.3
High (≥14.5 nmol/L)	32	50.8	16	57.1	16	45.7
Fatigue						
Mean (SD)	41.4 (8.5)		42.8 (8.4)		40.0 (8.7)	
Fatigued [≤42 (FACT-F)]	28	44.4	9	32.1	19	54.3
Not Fatigued [>42 (FACT-F)]	35	55.6	19	67.9	16	45.7
Aerobic exercise (min/week)						
Mean (SD)	52 (52)		40 (44)		62 (57)	
Not meeting AET guidelines	40	63.5	21	75.0	19	54.3
Meeting AET guidelines	23	36.5	7	25.0	16	45.7

*HIIT* high-intensity interval training, *No.* number, *SD* standard deviation, *VO*_*2*_*peak* peak aerobic exercise capacity, *ml* milliliter, *O*_*2*_ oxygen, *kg* kilograms, *BMI* body mass index, *min* minute, *nmol/L* nanomole per litre, *FACT-F* functional assessment of cancer therapy fatigue scale, *AET* aerobic exercise training

### Post-intervention effects

Table 2 reports the intervention effects on CRF and psychosocial function at post-intervention. Compared to UC, HIIT significantly improved CRF [adjusted between-group mean difference = 4.4; 95% CI, 1.5–7.3; *p* = 0.003] and self-esteem (*p* = 0.029) but not depression, anxiety, stress, or sleep quality. Table 3 reports the intervention effects on HRQoL. Compared to UC, HIIT significantly improved MCS (*p* = 0.034), vitality (*p* = 0.001), social functioning (*p* = 0.011), general health (*p* = 0.016), and role-physical (*p* = 0.048), and mental health (*p* = 0.054). No significant effects were noted for physical functioning, bodily pain, role-emotional, or PCS.

**Table 2:** Effects of 12 weeks of HIIT on CRF and psychosocial functioning at post-intervention in TCS

Measure	Group	No.	Baseline		Post-intervention				Between-group difference	
			Mean	SD	Mean	SD	Adj. Mean^a^	SE	Adj. Mean^a^ (95% CI)	*p*
CRF	Control	27	42.8	8.4	41.7	8.9	40.6	1.1	4.4 (1.5 to 7.3)	0.003
	Exercise	35	40.0	8.7	44.2	7.0	45.0	0.9		
Depression	Control	27	4.5	4.4	4.0	3.8	4.2	0.5	−0.2 (−1.6 to 1.3)	0.81
	Exercise	35	5.3	4.7	4.2	3.3	4.0	0.5		
Anxiety	Control	27	16.2	4.7	17.4	5.9	18.0	0.9	−1.6 (−3.9 to 0.8)	0.19
	Exercise	35	18.6	5.5	16.9	4.2	16.4	0.8		
Stress	Control	27	16.3	9.1	17.0	8.5	18.1	1.0	−1.7 (−4.4 to 1.0)	0.22
	Exercise	35	19.7	8.5	17.3	6.7	16.4	0.9		
Self-esteem	Control	27	36.0	4.8	35.0	5.0	33.7	0.6	1.8 (0.2 to 3.4)	0.029
	Exercise	35	32.5	5.5	34.5	4.1	35.5	0.5		
Sleep quality	Control	27	3.2	2.5	3.2	2.9	3.6	0.3	−0.6 (−1.4 to 0.2)	0.15
	Exercise	35	3.9	2.0	3.3	2.0	3.0	0.3		

*HIIT* high-intensity aerobic interval training, *CRF* cancer-related fatigue, *TCS* testicular cancer survivors, *No.* number, *SD* standard deviation, *Adj.* adjusted, *SE* standard error, *CI* confidence interval.

^a^All follow-up and between-group difference values were adjusted for baseline value of the outcome, age, treatment exposure, and time since treatment

**Table 3:** Effects of 12 weeks of HIIT on HRQoL at post-intervention in TCS

Measure	Group	No.	Baseline		Post-intervention				Between-group difference	
			Mean	SD	Mean	SD	Adj. Mean^a^	SE	Adj. Mean^a^ (95% CI)	*p*
MCS	Control	27	50.0	6.7	47.3	9.3	46.9	1.4	3.9 (0.3 to 7.5)	0.034
	Exercise	35	48.0	9.4	50.4	6.3	50.8	1.2		
PCS	Control	27	53.4	5.9	54.1	7.0	53.2	0.9	1.1 (−1.2 to 3.5)	0.34
	Exercise	35	51.0	7.3	53.6	5.2	54.3	0.8		
Physical functioning	Control	27	54.1	4.1	54.8	3.7	54.4	0.7	0.3 (−1.5 to 2.1)	0.77
	Exercise	35	52.6	6.9	54.4	4.4	54.7	0.6		
Role-physical	Control	27	53.9	5.3	53.7	6.0	52.3	0.8	2.2 (0.02 to 4.3)	0.048
	Exercise	35	50.0	10.9	53.4	7.2	54.5	0.7		
Bodily pain	Control	27	53.5	8.4	52.8	7.9	51.9	1.1	1.3 (−1.8 to 4.3)	0.41
	Exercise	35	50.7	7.0	52.5	7.3	53.2	1.0		
General health	Control	27	51.0	8.1	50.9	8.3	49.8	1.0	3.2 (0.6 to 5.8)	0.016
	Exercise	35	48.5	7.5	52.2	6.8	53.0	0.8		
Vitality	Control	27	52.9	9.6	50.8	8.9	50.2	1.2	5.4 (2.2 to 8.5)	0.001
	Exercise	35	51.2	8.1	55.0	7.8	55.5	1.0		
Social functioning	Control	27	44.2	5.0	42.6	7.4	42.1	0.9	3.3 (0.8 to 5.8)	0.011
	Exercise	35	43.0	7.6	45.0	5.1	45.4	0.8		
Role-emotional	Control	27	52.0	6.7	51.1	7.7	50.5	1.2	1.5 (−1.7 to 4.7)	0.36
	Exercise	35	49.5	9.0	51.4	6.5	52.0	1.0		
Mental health	Control	27	53.7	6.4	51.0	8.3	50.5	1.2	3.2 (−0.1 to 6.5)	0.054
	Exercise	35	50.9	9.1	53.4	5.2	53.7	1.1		

*HIIT* high-intensity aerobic interval training, *HRQoL* health-related quality of life, *TCS* testicular cancer survivors, *No.* number, *SD* standard deviation, *Adj.* adjusted, *SE* standard error, *CI* confidence interval, *MCS* mental component score, *PCS* physical component score.

^a^All follow-up and between-group difference values were adjusted for baseline value of the outcome, age, treatment exposure, and time since treatment

### Three month follow-up effects

**Fig. 2:**
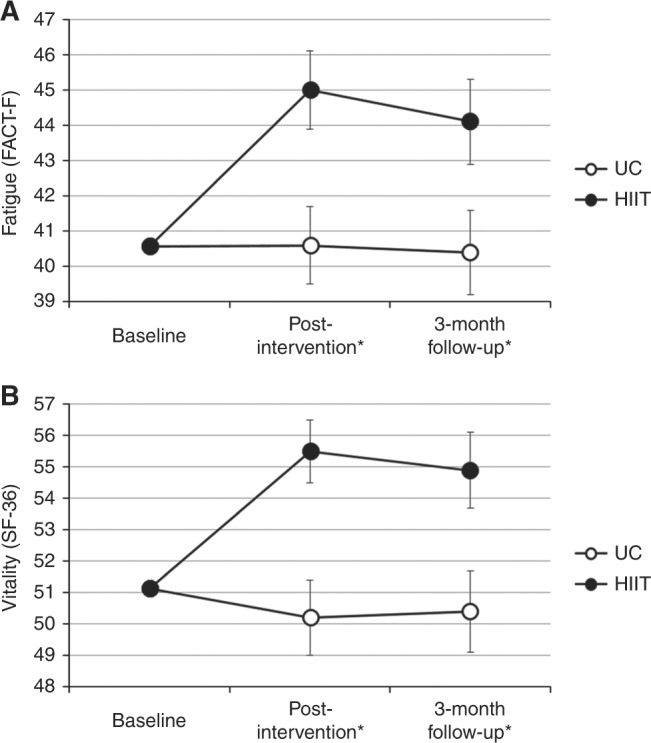
Effects of 12 weeks of HIIT on **a**) CRF and **b**) vitality at post-intervention and 3-month follow-up in TCS. HIIT high-intensity aerobic interval training, CRF cancer-related fatigue, TCS testicular cancer survivors, FACT-F functional assessment of cancer therapy fatigue scale, UC usual care, SF-36 short form 36. ^*^ Post-intervention and 3-month follow-up difference values were adjusted for baseline value of the outcome, age, treatment exposure, and time since treatment

At 3-month follow-up, HIIT maintained its significant effect on CRF (*p* = 0.031; Fig. 2A) and vitality (*p* = 0.015; Fig. 2B). There were no other significant between-group differences for any other psychosocial or HRQoL outcomes at 3-month follow-up. Tables S1 and S2 in the online supplement provide full 3-month follow-up results.

### Exploratory mediation

Table S3 in the online supplement reports the linear regression analyses for change in VO_2peak_ as a potential mediator of changes in PROs. At post-intervention, we found evidence that changes in VO_2peak_ mediated changes in MCS (*p* < 0.05), vitality (*p* < 0.10), and mental health (*p* < 0.05). At 3-month follow-up, we found evidence that changes in VO_2peak_ mediated changes in CRF (*p* < 0.10) and vitality (*p* < 0.05).

### Exploratory moderation

**Fig. 3:**
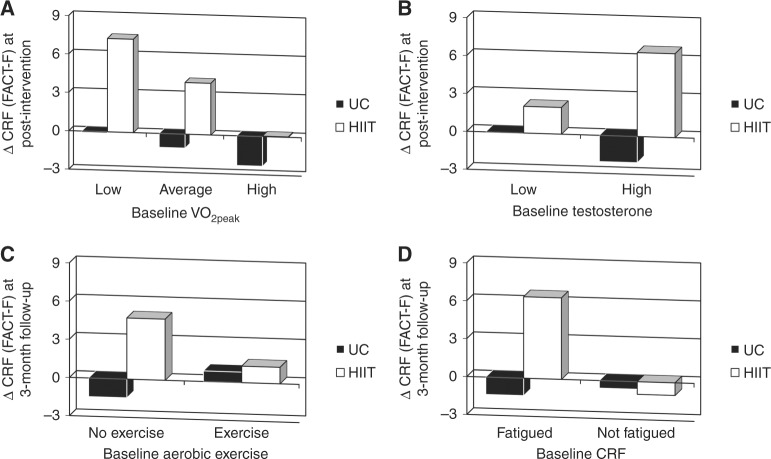
Moderator effects of baseline **a)** VO_2peak_ and **b)** testosterone on CRF at post-intervention and baseline **c)** aerobic exercise and **d)** CRF on CRF at 3-month follow-up. VO_2peak_ peak aerobic exercise capacity, CRF cancer-related fatigue, FACT-F functional assessment of cancer therapy fatigue scale, UC usual care, HIIT high-intensity aerobic interval training

Figure 3 and Table S4 (online supplement) report the effects of key clinical variables as moderators of changes in CRF at post-intervention and 3-month follow-up. We found evidence that post-intervention changes in CRF were moderated by baseline VO_2peak_ (*p*_interaction_ = 0.09) and baseline testosterone levels (*p*_interaction_ = 0.04). Changes in CRF at 3-month follow-up were moderated by baseline aerobic exercise (*p*_interaction_ = 0.08) and baseline fatigue (*p*_interaction_ = 0.04).

## Discussion

As hypothesised, HIIT caused a significant and clinically meaningful post-intervention improvement in CRF. Although no previous exercise studies in TCS exist for comparison purposes, the HIIT-related improvement in CRF of 4.4 points exceeded the 3-point clinically important difference (CID) for the FACT-F^36^ and the standardised effect size of *d* = 0.52 represents a medium effect size. This effect size is larger than the 0.22–0.30 reported in recent meta-analyses of aerobic exercise and fatigue in cancer survivors^12^. The larger improvement in CRF observed in the HIITTS trial may be related to changes in physiologic [e.g., increased mitochondrial biogenesis or oxidative capacity^37,38^] and psychosocial [e.g., self-efficacy^39^] variables, which may be less impacted by moderate-intensity continuous aerobic exercise^12^. Randomised controlled trials are needed to directly compare HIIT exercise to moderate-intensity continuous exercise for CRF in cancer survivors.

Our exploratory moderation findings suggest that the effects of HIIT on CRF were significantly larger for TCS, who at baseline had lower fitness, higher testosterone, an inactive lifestyle, and clinical fatigue. These findings confirm the expected but often ignored observation that exercise interventions designed to improve CRF are most effective in survivors who are not exercising, have low fitness, and clinical levels of fatigue. In these subgroups, exercise effects on CRF were twice the CID on the FACT-F (>6 points) and substantially larger than the small effects reported in meta-analyses^12^. These data suggest that properly targeted HIIT-based exercise interventions may have profound effects on CRF in cancer survivors.

The moderation of exercise effects by baseline testosterone is novel and suggests a possible precision medicine approach to exercise oncology for PROs. Our findings are aligned with previous research that low testosterone levels may be an important determinant of physical and psychosocial health in cancer survivors including TCS^6,40^. The positive relationship between functional outcomes (e.g., VO_2peak_ and muscular strength) and serum testosterone concentrations is well established in healthy and clinical populations (e.g., heart failure), and testosterone supplementation in hypogonadal males causes significant improvements in VO_2peak_ and muscular strength^41,42^. However, whether testosterone supplementation would similarly benefit TCS remains unclear. Randomised controlled trials comparing exercise plus testosterone supplementation to exercise alone in TCS with low testosterone would provide a definitive answer to this question.

The small-to-moderate HIIT-related improvement in self-esteem (*d* = 0.35) is consistent with previous exercise-oncology research (*d* = 0.25–0.55)^18,43^. Our finding that change in VO_2peak_ does not mediate improvements in self-esteem is also consistent with previous research^18^ and suggests that the observed improvement in self-esteem may be related to changes in psychosocial constructs, such as self-efficacy, positive feedback from others, or a sense of accomplishment^39^.

Contrary to other exercise-oncology trials^13^, HIIT did not improve several psychosocial outcomes including depression, anxiety, stress, and sleep quality. The lack of observed effect may be attributable to the self-selection bias of TCS with relatively normal psychosocial functioning for these outcomes. Future exercise trials should target subgroups of TCS with baseline psychosocial distress.

As hypothesised, HIIT caused post-intervention improvements in multiple HRQoL domains, with moderate effects across mental health-related domains [i.e., MCS (*d* = 0.49), vitality (*d* = 0.61), social functioning (*d* = 0.52), and mental health (*d* = 0.41)] and small-to-moderate effects across physical health-related domains [i.e., role-physical (*d* = 0.27) and general health (*d* = 0.41)]; all of which exceeded the 2-3-point CID threshold for these scales (Maruish 2011)^31^. Our findings indicated stronger and more consistent effects on mental HRQoL; whereas, previous exercise-oncology research has reported stronger and more consistent effects on physical HRQoL domains^14^. Our finding that VO_2peak_ partially mediated the improvements in mental HRQoL domains was unexpected given that previous studies report changes in VO_2peak_ being strongly associated with improvements in physical, but not mental HRQoL domains^18,20^. Several factors may contribute to these findings including the nature of HRQoL deficits reported by TCS [i.e., greater mental HRQoL impairment than physical^8^] and the intervention effects on CRF^19^. Between 15% and 17% of TCS report feeling less masculine because of their surgery or treatments^8,44,45^; and, it is possible that by engaging in activities perceived to be masculine (e.g., physically demanding exercise), TCS may achieve improved psychosocial function and mental HRQoL. Further research is required to assess the relative, and perhaps synergistic, contributions of key mediators of the observed benefits of HIIT on HRQoL in TCS.

Interestingly, the HIIT-related improvements in CRF and vitality persisted at 3-month follow-up. The protracted and clinically meaningful^31,36^ benefits of HIIT on these fatigue-related outcomes [i.e., CRF (*d* = 0.43) and vitality (*d* = 0.48)] is surprising given that exercise-oncology studies have typically shown that group differences dissipate with the discontinuation of the intervention^20,46^. One notable difference in our trial is that our exercise crossover in the UC group did not occur until after the 3-month follow-up, whereas previous comparisons of longer term follow-up in exercise-oncology trials have often been confounded by a post-intervention crossover^20,46,47^. If confirmed, HIIT may be key treatment strategy to combat long-term fatigue-related deficits in TCS.

Overall, the HIIT-related improvement in VO_2peak_ mediated the observed changes in half of our significantly improved PROs. These findings have direct exercise prescription implications for future research and clinical practice, providing preliminary evidence that improving VO_2peak_ (or related physiological factors) may be important for improving CRF and related HRQoL in TCS.

The overall strengths and limitations of the HIITTS trial have been described elsewhere^17^. Briefly, we conducted the first randomised aerobic exercise trial in TCS with virtually 100% adherence and trivial loss-to-follow-up that produced substantial improvements in VO_2peak_ and CVD risk. Additional strengths in the present report include providing the first randomised data examining the effects of HIIT on PROs in TCS, the use of well-validated measures of PROs, the collection of 3-month follow-up data unconfounded by an exercise crossover, the examination of VO_2peak_ as a mediator of changes in PROs, and the exploration of clinically useful moderators. Previously reported limitations of the overall trial include no outcome assessor blinding, the low initial response rate, a limited follow-up period, and variable time since diagnosis. Additional limitations related to our PRO findings include the smaller sample size (and potential for self-selection bias), the loss-to-follow-up at 3-months, the imbalance of clinical fatigue between groups at baseline, the recruitment of TCS without specific psychosocial or HRQoL deficits, the lack of an attention control comparison group, and the exploratory nature of our mediation and moderator analyses. Although it is also possible that the potential self-selection bias may have resulted in an overestimation of the feasibility of the intervention, our ≤ 98% attendance/adherence data suggest that at least some TCS are able to integrate HIITT into full-time academic and employment schedules.

In conclusion, the HIITTS trial provides the first randomised evidence that a 12-week HIIT program improves CRF, self-esteem, and multiple domains of mental and physical HRQoL in TCS. Moreover, improvements in many of the PROs were partially mediated by VO_2peak_. In exploratory analyses, we found that the effects of HIIT on CRF were significantly larger for TCS who at baseline had lower fitness, higher testosterone, an inactive lifestyle, and clinical fatigue. Finally, the near-perfect adherence to our HIIT prescription suggests that HIIT is a well-tolerated and time-efficient exercise option for TCS. Our findings have important clinical implications for the management of TCS and identify opportunities for targeted exercise interventions designed to optimise improvements in PROs in TCS. Additional exercise research in this understudied survivor population with unmet needs is warranted.

## Electronic supplementary material

Figure S1

HIITTS - PRO - Online Supplement

## Availability of data & material

Study data is available upon request.

## References

[1] Curado, M et al. *Cancer Incidence In Five Continents*. (IARC Press, Lyon, 2007). .

[2] Miller KD (2016). Cancer treatment and survivorship statistics, 2016. CA Cancer J. Clin..

[3] Feldman DR, Bosl GJ, Sheinfeld J, Motzer RJ (2008). Medical treatment of advanced testicular cancer. JAMA.

[4] Haugnes HS (2012). Long-term and late effects of germ cell testicular cancer treatment and implications for follow-up. J. Clin. Oncol..

[5] Christensen JF, Bandak M, Campbell A, Jones LW, Hojman P (2015). Treatment-related cardiovascular late effects and exercise training countermeasures in testicular germ cell cancer survivorship. Acta Oncol..

[6] Sprauten M (2015). Chronic fatigue in 812 testicular cancer survivors during long-term follow-up: increasing prevalence and risk factors. Ann. Oncol..

[7] Dahl AA (2005). Study of anxiety disorder and depression in long-term survivors of testicular cancer. J. Clin. Oncol..

[8] Smith AB (2016). The prevalence, severity, and correlates of psychological distress and impaired health-related quality of life following treatment for testicular cancer: a survivorship study. J. Cancer Surviv.

[9] Mock V (2000). NCCN practice guidelines for cancer-related fatigue. Oncology.

[10] Orre IJ (2008). Chronic cancer-related fatigue in long-term survivors of testicular cancer. J. Psychosom. Res.

[11] Oechsle K (2016). Symptom burden in long-term germ cell tumour survivors. Support Care Cancer.

[12] Mustian KM (2017). Comparison of pharmaceutical, psychological, and exercise treatments for cancer-related fatigue: a meta-analysis. JAMA Oncol..

[13] Brown JC (2012). The efficacy of exercise in reducing depressive symptoms among cancer survivors: a meta-analysis. PloS ONE.

[14] Mishra SI, Scherer RW, Snyder C, Geigle P, Gotay C (2014). Are exercise programs effective for improving health-related quality of life among cancer survivors? A systematic review and meta-analysis. Oncol. Nurs. Forum.

[15] Thorsen L (2005). The association between self-reported physical activity and prevalence of depression and anxiety disorder in long-term survivors of testicular cancer and men in a general population sample. Support Care Cancer.

[16] Fung C (2017). Multi-institutional assessment of adverse health outcomes among North American testicular cancer survivors after modern cisplatin-based chemotherapy. J. Clin. Oncol..

[17] Adams SC (2017). Effects of high-intensity aerobic interval training on cardiovascular disease risk in testicular cancer survivors: a phase II randomised controlled trial. Cancer.

[18] Courneya KS (2003). Randomised controlled trial of exercise training in postmenopausal breast cancer survivors: Cardiopulmonary and quality of life outcomes. J. Clin. Oncol..

[19] Buffart LM (2013). Fatigue mediates the relationship between physical fitness and quality of life in cancer survivors. J. Sci. Med Sport.

[20] Courneya KS (2009). Randomised controlled trial of the effects of aerobic exercise on physical functioning and quality of life in lymphoma patients. J. Clin. Oncol..

[21] Rognmo O, Hetland E, Helgerud J, Hoff J, Slordahl SA (2004). High intensity aerobic interval exercise is superior to moderate intensity exercise for increasing aerobic capacity in patients with coronary artery disease. Eur. J. Cardiovasc Prev. Rehabil..

[22] Mezzani A (2013). Aerobic exercise intensity assessment and prescription in cardiac rehabilitation: a joint position statement of the European Association for Cardiovascular Prevention and Rehabilitation, the American Association of Cardiovascular and Pulmonary Rehabilitation and the Canadian Association of Cardiac Rehabilitation. Eur. J. Prev. Cardiol..

[23] Godin G, Shephard RJ (1985). A simple method to assess exercise behavior in the community. Can. J. Appl. Sport Sci..

[24] Schmitz KH (2010). American College of Sports Medicine roundtable on exercise guidelines for cancer survivors. Med Sci. Sports Exerc.

[25] Yellen SB, Cella DF, Webster K, Blendowski C, Kaplan E (1997). Measuring fatigue and other anemia-related symptoms with the Functional Assessment of Cancer Therapy (FACT) measurement system. J. Pain. Symptom Manag..

[26] Kohout FJ, Berkman LF, Evans DA, Cornoni-Huntley J (1993). Two shorter forms of the CES-D (Center for Epidemiological Studies Depression) depression symptoms index. J. Aging Health.

[27] Spielberger, C. D, Gorsuch, R. L, Lushene, R, Vagg, P. R. & Jacobs, G. A. *Manual For The State-trait Anxiety Inventory*. (Consulting Psychologists Press, Palo Alto, 1983).

[28] Cohen S, Kamarck T, Mermelstein R (1983). A global measure of perceived stress. J. Health Soc. Behav..

[29] Rosenberg, M *Society And The Adolescent Self-image.* Vol.11 (Princeton University Press, Princeton, 1965).

[30] Beck SL, Schwartz AL, Towsley G, Dudley W, Barsevick A (2004). Psychometric evaluation of the Pittsburgh Sleep Quality Index in cancer patients. J. Pain. Symptom Manag..

[31] Ware, J. E et al. *User’s Manual for the SF-36v2 Health Survey.* 2^nd^ edn. (Quality Metric Incorporated, Lincoln, 2011).

[32] MacKinnon, D. P *Introduction To Statistical Mediation Analysis*. (Lawrence Erlbaum Associates, New York, 2008).

[33] Hayes, A. F* Introduction To Mediation, Moderation, And Conditional Process Analysis: A Regression-based Approach*. 2^nd^ edn. (Guilford Press, New York, 2013).

[34] Preacher KJ, Hayes AF (2004). SPSS and SAS procedures for estimating indirect effects in simple mediation models. Behav. Res Methods Instrum. Comput..

[35] Borm GF, Fransen J, Lemmens WA (2007). A simple sample size formula for analysis of covariance in randomised clinical trials. J. Clin. Epidemiol..

[36] Cella D, Eton DT, Lai JS, Peterman AH, Merkel DE (2002). Combining anchor and distribution-based methods to derive minimal clinically important differences on the Functional Assessment of Cancer Therapy (FACT) anemia and fatigue scales. J. Pain. Symptom Manag..

[37] Daussin FN (2008). Effect of interval versus continuous training on cardiorespiratory and mitochondrial functions: relationship to aerobic performance improvements in sedentary subjects. Am. J. Physiol. Regul. Integr. Comp. Physiol..

[38] Wisloff U (2007). Superior cardiovascular effect of aerobic interval training versus moderate continuous training in heart failure patients: A randomised study. Circulation.

[39] Cormie P (2015). Can supervised exercise prevent treatment toxicity in patients with prostate cancer initiating androgen-deprivation therapy: a randomised controlled trial. BJU Int..

[40] Burney BO, Garcia JM (2012). Hypogonadism in male cancer patients. J. Cachex-. Sarcopenia Muscle.

[41] Bhasin S (2003). Testosterone supplementation for aging-associated sarcopenia. J. Gerontol. A Biol. Sci. Med Sci..

[42] Caminiti G (2009). Effect of long-acting testosterone treatment on functional exercise capacity, skeletal muscle performance, insulin resistance, and baroreflex sensitivity in elderly patients with chronic heart failure: a double-blind, placebo-controlled, randomised study. J. Am. Coll. Cardiol..

[43] Courneya KS (2007). Effects of aerobic and resistance exercise in breast cancer patients receiving adjuvant chemotherapy: a multicenter randomised controlled trial. J. Clin. Oncol..

[44] Rossen P, Pedersen AF, Zachariae R, von der Maase H (2012). Sexuality and body image in long-term survivors of testicular cancer. Eur. J. Cancer.

[45] van Basten JP (1996). Fantasies and facts of the testes. Br. J. Urol..

[46] Courneya KS (2007). Six-month follow-up of patient-rated outcomes in a randomised controlled trial of exercise training during breast cancer chemotherapy. Cancer Epidemiol. Biomark. Prev..

[47] Rogers LQ (2009). Physical activity and health outcomes three months after completing a physical activity behavior change intervention: persistent and delayed effects. Cancer Epidemiol. Biomark. Prev..

