# Supplementary figures and images for: Effects of high-intensity interval training on fatigue and quality of life in testicular cancer survivors
Source: Br J Cancer. 2018 May 8;118(10):1313–21. doi: 10.1038/s41416-018-0044-7 (PMC5959855; doi:10.1038/s41416-018-0044-7)

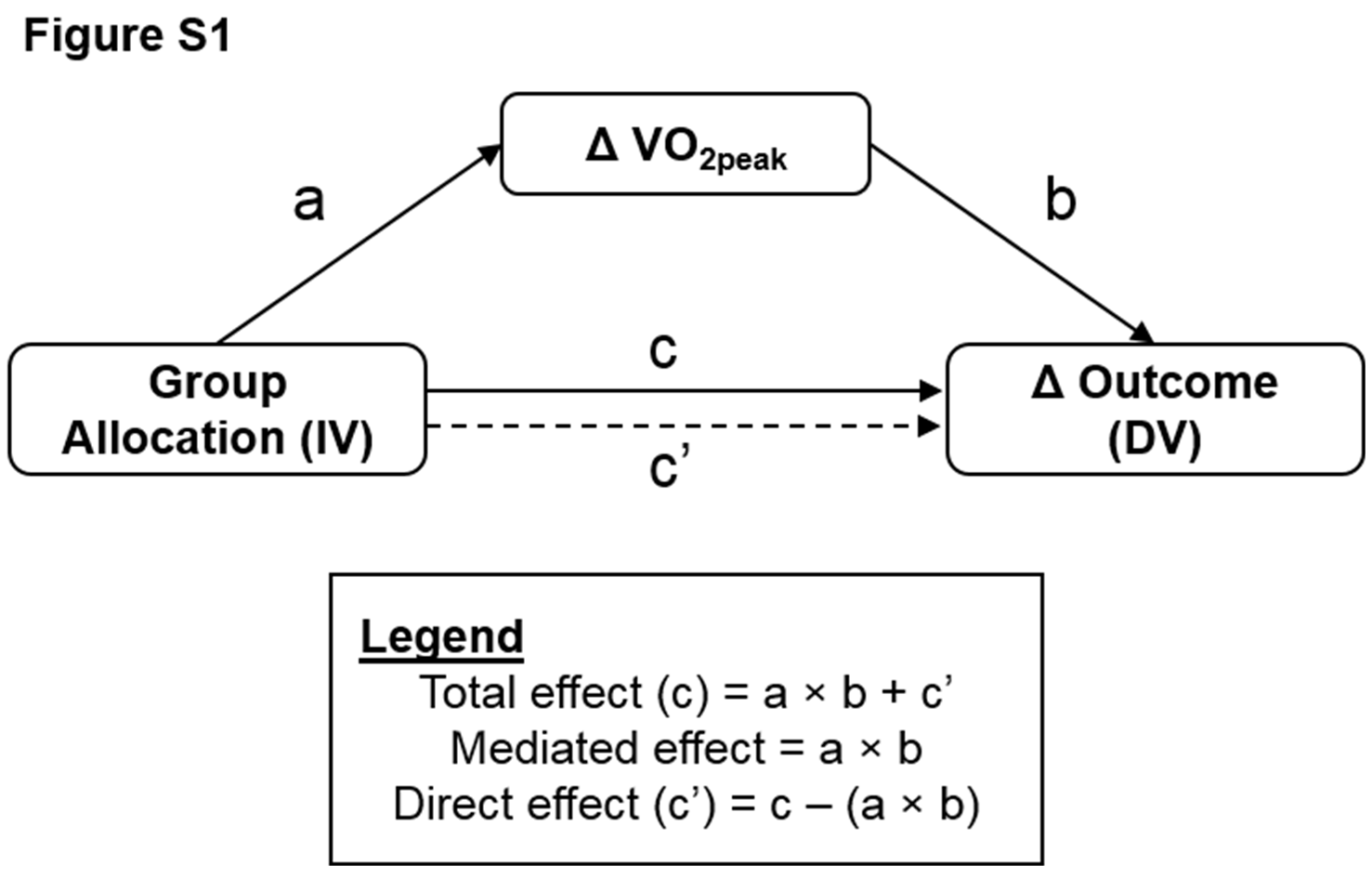

Supplement: Supplementary file 1 — Figure S1 [file 41416_2018_44_MOESM1_ESM.tif]
