# Supplementary material for: Effects of high-intensity interval training on fatigue and quality of life in testicular cancer survivors
Source: Br J Cancer. 2018 May 8;118(10):1313–21. doi: 10.1038/s41416-018-0044-7 (PMC5959855; doi:10.1038/s41416-018-0044-7)
Supplement: Supplementary file 2 — HIITTS - PRO - Online Supplement [file 41416_2018_44_MOESM2_ESM.docx]

**Online Supplementary Content**

Adams S.C. et al., Effects of high-intensity interval training on fatigue and quality of life in testicular cancer survivors.

**Table S1.** Effects of 12 weeks of HIIT on CRF and psychosocial functioning at 3-month follow-up in TCS

**Table S2.** Effects of 12 weeks of HIIT on HRQoL at 3-month follow-up in TCS

**Table S3.** Statistical test of change in VO_2peak_ as a mediator of the effects of 12 weeks of HIIT on change in PROs in TCS

**Table S4.** Effects of 12 weeks of HIIT on CRF at postintervention and 3-month follow-up in TCS by selected moderators

**Figure S1.** Mediation model: Δ VO2peak: change in peak aerobic fitness; IV: independent variable; DV: dependent variable.

| **Table S1: Effects of 12 weeks of HIIT on CRF and psychosocial functioning at 3-month follow-up in TCS** | | | | | | | | | | |
| --- | --- | --- | --- | --- | --- | --- | --- | --- | --- | --- |
| **Measure** | **Group** | **No,** | **Baseline** | | **3-month Follow-up** | | | | **Between-group difference** | |
|  |  |  | **Mean** | **SD** | **Mean** | **SD** | **Adj. Mean^*^** | **SE** | **Adj. Mean^*^ (95% CI)** | ***p*** |
| CRF | Control | 23 | 42.8 | 8.4 | 41.1 | 10.2 | 40.4 | 1.2 | 3.7 (0.4 to 7.1) | 0.031 |
|  | Exercise | 29 | 40.0 | 8.7 | 43.6 | 7.3 | 44.1 | 1.1 |  |  |
| Depression | Control | 23 | 4.6 | 4.7 | 5.6 | 5.2 | 5.7 | 0.6 | -1.2 (-2.9 to 0.5) | 0.17 |
|  | Exercise | 29 | 4.8 | 4.1 | 4.6 | 4.8 | 4.5 | 0.6 |  |  |
| Anxiety | Control | 23 | 16.5 | 4.9 | 18.7 | 4.0 | 19.1 | 0.8 | -1.3 (-3.5 to 0.9) | 0.25 |
|  | Exercise | 29 | 17.9 | 4.4 | 18.0 | 4.2 | 17.8 | 0.7 |  |  |
| Stress | Control | 23 | 16.5 | 9.3 | 17.6 | 9.0 | 18.7 | 1.1 | -2.4 (-5.4 to 0.7) | 0.12 |
|  | Exercise | 29 | 18.8 | 7.5 | 17.1 | 7.3 | 16.3 | 1.0 |  |  |
| Self-esteem | Control | 23 | 35.7 | 5.0 | 35.0 | 4.7 | 33.7 | 0.6 | 1.0 (-0.5 to 2.6) | 0.19 |
|  | Exercise | 29 | 33.2 | 4.5 | 33.8 | 4.8 | 34.8 | 0.5 |  |  |
| Sleep | Control | 23 | 3.4 | 2.6 | 3.3 | 2.9 | 3.6 | 0.4 | -0.7 (-1.8 to 0.4) | 0.19 |
|  | Exercise | 29 | 3.9 | 2.1 | 3.0 | 2.0 | 2.8 | 0.4 |  |  |

**Notes:** HIIT: high-intensity aerobic interval training; CRF: cancer-related fatigue; TCS: testicular cancer survivors; No.: number; SD: standard deviation; Adj.: adjusted; SE: standard error; CI: confidence interval

^*^ all follow-up and between-group difference values were adjusted for baseline value of the outcome, age, treatment exposure, and time since treatment.

| **Table S2: Effects of 12 weeks of HIIT on HRQoL at 3-month follow-up in TCS** | | | | | | | | | | |  |
| --- | --- | --- | --- | --- | --- | --- | --- | --- | --- | --- | --- |
| **Measure** | **Group** | **No.** | **Baseline** | | **3-month Follow-up** | | | | **Between-group difference** | |  |
|  |  |  | **Mean** | **SD** | **Mean** | **SD** | **Adj. Mean^*^** | **SE** | **Adj. Mean^*^ (95% CI)** | ***p*** | |
| MCS | Control | 23 | 49.5 | 6.9 | 47.9 | 9.6 | 47.8 | 1.4 | 1.3 (-2.5 to 5.0) | 0.51 | |
|  | Exercise | 29 | 49.3 | 7.5 | 49.1 | 8.6 | 49.1 | 1.2 |  |  | |
| PCS | Control | 23 | 53.2 | 6.1 | 52.9 | 6.8 | 52.2 | 1.1 | 2.2 (-0.8 to 5.2) | 0.14 | |
|  | Exercise | 29 | 50.7 | 7.8 | 53.8 | 5.6 | 54.4 | 1.0 |  |  | |
| Physical functioning | Control | 23 | 53.7 | 4.3 | 53.4 | 4.8 | 53.2 | 1.0 | 0.8 (-1.9 to 3.5) | 0.56 | |
|  | Exercise | 29 | 52.6 | 7.3 | 53.8 | 5.2 | 54.0 | 0.9 |  |  | |
| Role-physical | Control | 23 | 53.6 | 5.6 | 52.9 | 6.6 | 52.1 | 1.1 | 1.7 (-1.4 to 4.8) | 0.28 | |
|  | Exercise | 29 | 50.2 | 11.1 | 53.1 | 7.2 | 53.7 | 1.0 |  |  | |
| Bodily pain | Control | 23 | 53.1 | 8.9 | 52.0 | 8.9 | 51.2 | 1.4 | 3.0 (-0.8 to 6.7) | 0.12 | |
|  | Exercise | 29 | 50.5 | 6.8 | 53.4 | 7.4 | 54.1 | 1.2 |  |  | |
| General health | Control | 23 | 51.3 | 8.5 | 50.5 | 9.7 | 50.0 | 1.2 | 2.5 (-0.7 to 5.6) | 0.12 | |
|  | Exercise | 29 | 49.4 | 7.6 | 51.5 | 7.1 | 52.1 | 1.0 |  |  | |
| Vitality | Control | 23 | 52.0 | 10.0 | 50.6 | 10.9 | 50.4 | 1.3 | 4.5 (0.9 to 8.0) | 0.015 | |
|  | Exercise | 29 | 51.7 | 8.6 | 54.7 | 9.0 | 54.9 | 1.2 |  |  | |
| Social functioning | Control | 23 | 44.1 | 5.2 | 43.2 | 7.7 | 43.1 | 1.1 | 0.9 (-1.9 to 3.8) | 0.51 | |
|  | Exercise | 29 | 43.8 | 6.8 | 43.9 | 4.9 | 44.0 | 0.9 |  |  | |
| Role-emotional | Control | 23 | 51.7 | 7.1 | 50.0 | 9.5 | 49.6 | 1.7 | 0.4 (-4.1 to 5.0) | 0.85 | |
|  | Exercise | 29 | 50.4 | 7.8 | 49.7 | 8.9 | 50.0 | 1.5 |  |  | |
| Mental health | Control | 23 | 53.1 | 6.7 | 51.8 | 8.3 | 51.6 | 1.2 | 1.3 (-2.0 to 4.6) | 0.44 | |
|  | Exercise | 29 | 52.2 | 7.0 | 52.7 | 6.8 | 52.9 | 1.1 |  |  | |

**Notes:** HIIT: high-intensity aerobic interval training; HRQoL: health related quality of life; TCS: testicular cancer survivors; No.: number; SD: standard deviation; Adj.: adjusted; SE: standard error; CI: confidence interval; MCS: mental component score; PCS: physical component score

^*^ all follow-up and between-group difference values were adjusted for baseline value of the outcome, age, treatment exposure, and time since treatment.

| **Table S3: Statistical test of change in VO_2peak_ as a mediator of the effects of 12 weeks of HIIT on change in PROs in TCS** | | | | | |
| --- | --- | --- | --- | --- | --- |
| **Outcome ^c^** | **Total Effect^a^:**  **Group 🡪 Δ Outcome** | **Direct Effect^a,b^:**  **Group 🡪 Δ Outcome** | **Indirect Effect^a^:**  **Group 🡪 Δ VO_2peak_** | **Indirect Effect^a^:**  **Δ VO_2peak_ 🡪 Outcome** | **Mediation Effect^a^:** |
|  | **(path c)** | **(path c’)** | **(path a)** | **(path b)** | **(a x b)** |
|  | **Estimate (95% CI)** | **Estimate (95% CI)** | **Estimate (95% CI)** | **Estimate (95% CI)** | **Estimate (95% CI)** |
| ***Postintervention*** | | | | | |
| CRF change | 4.1 (1.2; 7.1) **^**^** | 2.5 (-1.1; 6.1) | 4.0 (2.5; 5.5) **^**^** | 0.4 (-0.1;0.9) | 1.6 (-0.7; 3.9) |
| Self-Esteem change | 1.3 (-0.3; 3.0) | 0.5 (-1.5; 2.5) | 4.2 (2.6; 5.8) **^**^** | 0.2 (-0.1; 0.5) | 0.8 (-0.4; 2.1) |
| MCS change | 3.7 (-0.04; 7.4) ^*^ | 1.1 (-3.4; 5.6) | 3.9 (2.5; 5.4) **^**^** | 0.7 (-0.003; 1.3) ^*^ | **2.6 (0.2; 5.8) ^**^** |
| Role-Physical change | 1.5 (-0.4; 3.5) | 1.2 (-1.2; 3.7) | 4.0 (2.5; 5.6) **^**^** | 0.1 (-0.3; 0.4) | 0.3 (-1.0; 1.8) |
| General Health change | 3.1 (0.4; 5.8) **^**^** | 2.5 (-1.0; 5.9) | 4.3 (2.8; 5.8) **^**^** | 0.2 (-0.4; 0.7) | 0.7 (-1.2; 2.8) |
| Vitality change | 4.9 (1.7; 8.2) **^**^** | 2.4 (-1.5; 6.2) | 4.0 (2.5; 5.5) **^**^** | 0.6 (0.1; 1.2) **^**^** | **2.6 (-0.1; 5.4)^*^** |
| Social Function change | 2.7 (0.3; 5.2) **^**^** | 1.5 (-1.5; 4.5) | 4.0 (2.5; 5.5) **^**^** | 0.3 (-0.1; 0.8) | 1.2 (-0.6; 3.4) |
| Mental Health change | 2.4 (-0.8; 5.5) | -0.4 (-4.1; 3.4) | 4.0 (2.5; 5.5) **^**^** | 0.7 (0.1; 1.2) **^**^** | **2.7 (0.7; 5.8) ^**^** |
| ***3-month Follow-up*** | | | | | |
| CRF change | 3.0 (-0.3; 6.3) ^*^ | 0.5 (-3.5; 4.5) | 4.4 (2.7; 6.1) **^**^** | 0.6 (0.01; 1.1) **^**^** | **2.5 (-0.4; 5.4)^*^** |
| Vitality change | 4.1 (0.5; 7.8) **^**^** | 1.1 (-3.3; 5.6) | 4.4 (2.7; 6.1) **^**^** | 0.7 (0.1; 1.3) **^**^** | **3.0 (0.02; 6.4) ^**^** |

**Notes:** VO_2peak_: peak cardiorespiratory fitness; HIIT: high-intensity aerobic interval training; PROs: patient reported outcomes; TCS: testicular cancer survivors; Δ: change; CI: confidence interval; CRF: cancer-related fatigue; MCS: mental component score

^*^ p ≤ .10; ^**^ p < 0.05. Mediation effects with p < 0.10 are bolded.

^a^ Beta-weights are adjusted for baseline value of the outcome, baseline value of the mediator (VO_2peak_), age, treatment exposure, and time since treatment.

^b^ Adjusted for the mediator (change in VO_2peak_).

^c^ Total effect in mediation analyses is slightly different than the ANCOVA analyses because 1 participant was eliminated from the mediation analyses due to missing VO_2peak_ at postintervention.

| **Table S4: Effects of 12 weeks of HIIT on CRF at postintervention and 3-month follow-up in TCS by selected moderators** | | | | | | |
| --- | --- | --- | --- | --- | --- | --- |
|  | **Δ CRF at**  **Postintervention** | | **Adjusted Interaction Effect** | **Δ CRF at 3-month**  **Follow-up** | | **Adjusted Interaction Effect** |
| **Moderator** | **Control**  **(*n=28*)** | **HIIT**  **(*n=35*)** | **β (95% CI); p** | **Control**  **(*n=23*)** | **HIIT**  **(*n=29*)** | **β (95% CI); p** |
| **Baseline Age** | | | | | | |
| Younger | -1.5 | 3.6 | -0.10 (-6.67 to 6.48); 0.98 | -1.5 | 2.2 | -0.19 (-7.68 to 7.30); 0.96 |
| Older | -0.3 | 4.8 |  | 0.4 | 3.9 |  |
| **Baseline VO_2peak_** | | | | | | |
| Low | -0.1 | 7.3 | -3.79 (-8.21 to 0.64); 0.09 | -0.3 | 5.4 | -2.71 (-7.66 to 2.24); 0.28 |
| Average | -1.1 | 4.0 |  | -0.9 | 3.1 |  |
| High | -2.3 | 0.0 |  | -1.6 | 0.3 |  |
| **Baseline AET** | | | | | | |
| No Exercise | -1.4 | 5.7 | -4.28 (-9.98 to 1.42); 0.14 | -1.5 | 4.8 | -5.82 (-12.34 to 0.70); 0.08 |
| Exercise | -0.4 | 2.4 |  | 0.8 | 1.3 |  |
| **Treatment Exposure** | | | | | | |
| No Chemotherapy | -0.8 | 5.1 | -1.09 (-7.06 to 4.89); 0.72 | 0.0 | 3.2 | 3.02 (-4.52 to 10.56); 0.43 |
| Chemotherapy | -2.1 | 2.7 |  | -3.3 | 2.9 |  |
| **Baseline Testosterone** | | | | | | |
| Low | 0.0 | 2.1 | 6.59 (0.49 to 12.68); 0.04 | -1.0 | 0.8 | 5.00 (-2.01 to 12.01); 0.16 |
| High | -2.1 | 6.6 |  | -0.8 | 6.0 |  |
| **Baseline CRF** | | | | | | |
| Fatigued | 0.6 | 7.4 | -4.47 (-11.00 to 2.06); 0.18 | -1.4 | 6.4 | -8.21 (-15.91 to -0.51); 0.04 |
| Not Fatigued | -1.9 | 0.4 |  | -0.6 | -1.0 |  |

**Notes:** HIIT: high-intensity aerobic interval training; CRF: cancer-related fatigue; TCS: testicular cancer survivors; CI: confidence interval; AET: aerobic exercise behaviour

^*^ all follow-up and between-group difference values were adjusted for baseline value of the outcome, age, treatment exposure, and time since treatment.
